# Urinary 3-(3-Hydroxyphenyl)-3-hydroxypropionic Acid, 3-Hydroxyphenylacetic Acid, and 3-Hydroxyhippuric Acid Are Elevated in Children with Autism Spectrum Disorders

**DOI:** 10.1155/2016/9485412

**Published:** 2016-03-30

**Authors:** Xiyue Xiong, Dan Liu, Yichao Wang, Ting Zeng, Ying Peng

**Affiliations:** Maternal and Child Health Care Hospital of Hunan Province, Changsha 410008, China

## Abstract

Autism spectrum disorders (ASDs) are a group of mental illnesses highly correlated with gut microbiota. Recent studies have shown that some abnormal aromatic metabolites in autism patients are presumably derived from overgrown* Clostridium* species in gut, which may be used for diagnostic purposes. In this paper, a GC/MS based metabolomic approach was utilized to seek similar biomarkers by analyzing the urinary information in 62 ASDs patients compared with 62 non-ASDs controls in China, aged 1.5–7. Three compounds identified as 3-(3-hydroxyphenyl)-3-hydroxypropionic acid (HPHPA), 3-hydroxyphenylacetic acid (3HPA), and 3-hydroxyhippuric acid (3HHA) were found in higher concentrations in autistic children than in the controls (*p* < 0.001). After oral vancomycin treatment, urinary excretion of HPHPA (*p* < 0.001), 3HPA (*p* < 0.005), and 3HHA (*p* < 0.001) decreased markedly, which indicated that these compounds may also be from gut* Clostridium* species. The sensitivity and specificity of HPHPA, 3HPA, and 3HHA were evaluated by receiver-operating characteristic (ROC) analysis. The specificity of each compound for ASDs was very high (>96%). After two-regression analysis, the optimal area under the curve (AUC, 0.962), sensitivity (90.3%), and specificity (98.4%) were obtained by ROC curve of Prediction probability based on the three metabolites. These findings demonstrate that the measurements of the three compounds are strong predictors of ASDs and support the potential clinical utility for identifying a subgroup of ASDs subjects.

## 1. Introduction 

Autism spectrum disorders (ASDs) are neurodevelopmental disorders characterized by limited social interaction, abnormal use of language, and stereotypical behaviors, interests, and activities [1]. During the last decades, ASDs prevalence estimates have risen to as much as 113/10,000 children in the USA (2012) and 62/10,000 globally [2], corresponding to 1 : 88 and 1 : 161 children, respectively. Hence this once rare disease has now become one of the most frequent conditions in child neuropsychiatry and it has to be paid more attention to. ASDs's etiology and pathogenesis are not precisely known, although genetic and environmental factors have been proposed as the two primary causes of ASDs heritability estimates have shown a trend of decrease in a recent study [3], leaving sufficient room for environmental contributions to explain ASDs.

Among environmental factors possibly relevant to clinical feature, the overgrowth of unusual gut microbial species in a sizable subgroup of autistic patients is of great interest reported in several recent studies [4–8]. An excess of* Ruminococcus* and* Clostridium* species was initially reported in fecal samples from ASDs patients compared with the controls [4]. Parracho found a higher incidence of the* Clostridium histolyticum* group (*Clostridium* clusters I and II) in the fecal flora of 58 ASDs children compared to 10 healthy children. Interestingly, 12 unaffected siblings of ASDs probands displayed intermediate levels. Several members of the* C. histolyticum* group are known toxin producers which could lead to gut dysfunction [7]. Adams et al. found lower levels of bifidobacteria in 58 ASDs children compared to 39 controls. The growth of bifidobacteria may be inhibited by some unusual microbial species overgrown in gut, such as* Clostridium* species [8]. Additionally, recent studies have documented elevated concentrations of abnormal aromatic metabolites presumably derived from overgrown* Clostridium* species or other gut microbiota in the urine of autistic individuals [9–14]. In this study, to seek similar markers and further explore possible pathophysiological roles of gut microbiota in ASDs, we have developed a GC-MS based metabolomic approach for urine analysis in 62 autistic individuals and in 62 sex- and age-matched non-ASDs controls.

## 2. Material and Methods

### 2.1. Patient Recruitment and Sample Collection

This prospective study was approved by the Ethics Committee of Maternity and Child Care Hospital of Hunan Province. Informed consent was obtained from the parents of the patients. Sixty-two patients (48 males and 14 females aged from 1.5 to 7 years) previously diagnosed with ASDs and age/gender-matched non-ASDs controls (male 48, female 14) were obtained from Maternity and Child Care Hospital of Hunan Province. All the children with ASDs did not have a history of food restricted. The controls were excluded with mental retardation, verbal disorder, attention deficit hyperactivity disorder, and tics, and the ASDs cases were diagnosed according to DSM-IV diagnostic criteria. Children included in the study had no antianaerobic drug use history. Urine samples were collected into untreated vials during routine medical consultations, principally in the morning, and the exact time of collection was recorded. Each urine sample was aliquoted into 1.5 mL Eppendorf tubes and stored at −70°C immediately after collection until analysis.

### 2.2. Sample Pretreatment

The samples were pretreated as described in our previous work [15]. Briefly, urine samples were thawed at room temperature and centrifuged (at 3000 g) for 10 min and 100 *μ*L urine samples (contained 2.5 mmol/L creatinine) was first treated with 30.0 *μ*L urease (1.2 U/*μ*L) at 37°C for 30 min to remove interfering urea and then spiked with heptadecanoic acid (0.5 mg/mL, 50 *μ*L). Proteins, including the added urease, were precipitated with 800 *μ*L ethanol and removed after 15 min centrifugation (12000 r/min). Forty microliters of 0.04 mol/L hydroxylamine hydrochloride and 60 *μ*L of 0.05 mol/L Ba(OH)_2_ was added to the deproteinized solution and the mixture was then incubated at room temperature for 20 min. Subsequently, the mixture solution was evaporated to dryness, and the compounds in the dried residue were converted to TMS derivatives with 100 *μ*L of BSTFA/TMCS (100 : 1) and analyzed by GC/MS. More experimental details can be found in our patented technology (ZL 201210114246.2).

### 2.3. GC-MS Analysis

An Agilent GC-MS system (7890-5975C) was used to analyze the derivative samples. A sample (1 *μ*L) was injected with a split ratio of 50 : 1 into the GC and then separated with a fused silica HP-5 capillary column (30 m, 0.25 mm inside diameter, 0.25 *μ*m thickness of the inner liquid in the column). The chromatographic conditions were as follows. The injector temperature was set at 250°C. High purity nitrogen was used as carrier gas at a constant flow rate of 1.5 mL/min. The column temperature was initially kept at 60°C for 4 min, ramped to 320°C at 6.5°C/min, and then held for 10 min. The parameters of the mass spectrum were as follows. The interphase temperature and ion source temperature were 300°C and 230°C, respectively. Ions were generated by electronic impact (EI) at 70 eV. Masses were acquired from *m*/*z* 50 to 800. Drift of retention time of each peak was minimized by locking heptadecanoic acid at 36.00 min with retention time locking technology (RTL, Agilent). GC/MSD ChemStation Software was used for autoacquisition of GC total ion chromatograms (TICs) and fragmentation patterns. Each compound had a fragmentation pattern composed of a series of split molecular ions; the mass charge ratios and the abundance of which were compared with a standard mass chromatogram in the NIST (National Institute of Standards and Technology) mass spectra library by the ChemStation Software. Peaks with similarity index more than 70% were assigned compound names.

The chromatograms were subjected to noise reduction, and peaks with intensity higher than threefold of the ratio of signal to noise (S/N) were recorded prior to peak area integration. The relative intensity of each peak was normalized against that of the internal standard in GC/MS run. All known artifact peaks, such as peaks due to column bleed and BSTFA artifact peaks, were not considered in the final data analyses. Integrated peak areas of multiple derivative peaks belonging to the same compound were summed and considered as a single compound. Each sample was characterized by the same number of variables, and each of these variables was represented across all observations with the same sequence. Thus, a data matrix was generated by intensities of the commensal peaks from all samples to characterize the biochemical pattern of each sample. The resulting three-dimensional matrix consisting of peak indices (retention time (RT)-*m*/*z* pairs), sample names (observations), and normalized peak areas (variables) was exported for principal component analysis (PCA).

### 2.4. Statistical Analysis

After GC/MS analysis, each sample was represented by a GC/MS TIC, and ion peak areas of compounds were integrated. The peak area ratio of each compound to creatinine was calculated as the response. The results are expressed as ratios to the urinary creatinine concentration. Statistical analysis was used for the comparison of the metabolite levels to determine their significant differences between the ASDs group and the control group. The differentially expressed compounds with *p* values of <0.05 were considered to be statistically significant.

Principal component analysis (PCA) was used to differentiate the samples and performed by Mass Profiler Professional software (Agilent). All of the data from the differentially expressed compounds were used for constructing PCA models. The score plots of the first three principal components allowed the visualization of data and comparison of samples between the ASDs and control group. The classification performance (specificity and sensitivity) was assessed by the area under the curve (AUC) of the receiver-operating characteristic (ROC) curves.

## 3. Results and Discussion

### 3.1. Metabolomic Profiling of Urine Samples

**Figure 1:**
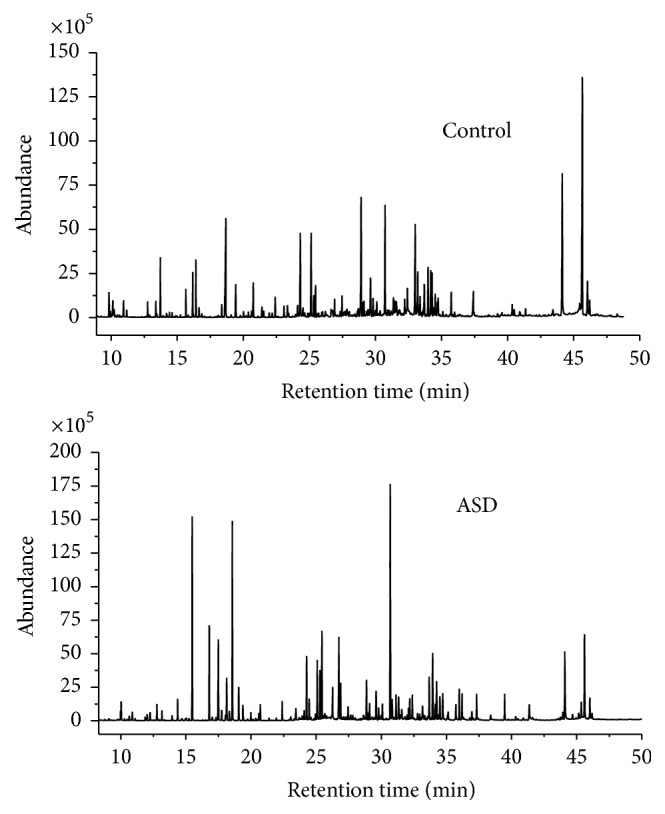
Representative GC/MS total ion chromatograms of the samples from the control group and the ASDs group after chemical derivatization.

Representative GC/MS TIC chromatograms of urine samples from the ASDs group and the control group are displayed in Figure 1. The majority of the peaks in the chromatograms were identified as endogenous metabolites by the NIST mass spectra library, including amino acids, organic acids, carbohydrates, amides, and fatty acids. Table S1 in electronic Supplementary Material (see the Supplementary Material available online at http://dx.doi.org/10.1155/2016/9485412) shows the 96 signals, which could be autoidentified by the NIST library through comparing their fragmentation patterns composed of all the fragment ions. Peaks which could not be identified by the NIST library are not listed. The first three fragment-ion *m*/*z* values with the highest abundance within each fragmentation pattern and the matching percentage to the NIST library are listed in Table S1.

### 3.2. Pattern Recognition and Function Analysis

**Figure 2:**
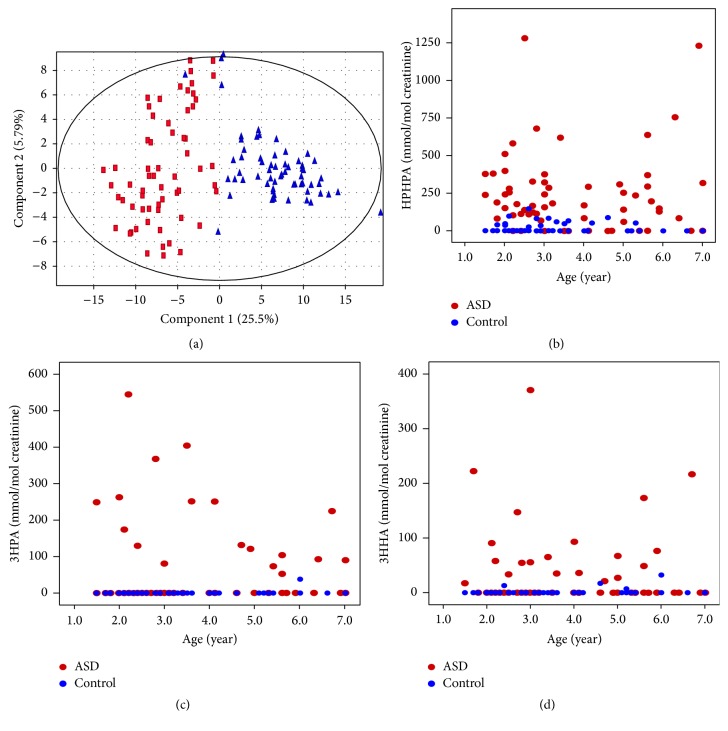
PCA scores plot and distributions of the three compounds. (a) PCA scores plot of the ASDs group from the control group. Triangles represent the ASDs cases and squares represent the control cases. (b) Distribution of urinary HPHPA concentration by age between ASDs and control subjects. (c) Distribution of urinary 3HPA concentration by age between ASDs and control subjects. (d) Distribution of urinary 3HHA concentration by age between ASDs and control subjects.

After normalization of data using creatinine as internal standard, a number of differentially expressed compounds with *p* values of <0.05 were selected by statistical analysis to construct a PCA model for assessment of the clustering of the ASDs group and the control group. The PCA scores plot showed a clear separation of the two groups besides only a few ASDs cases (Figure 2(a)), which could be explained by the fact that the etiology and pathogenesis of these cases are probably caused by genetic factors. According to the previously reported studies, elevated concentration of 3-(3-hydroxyphenyl)-3-hydroxypropionate (HPHPA) may be the catabolism product of phenylalanine by* Clostridium* species [9]. Interestingly, besides HPHPA, we also found two aromatic metabolites 3-hydroxyphenylacetic acid (3HPA) and 3-hydroxyhippuric acid (3HHA) among these differentially expressed compounds. The three compounds were further qualitatively analyzed by comparing their retention time and fragment-ion of the chromatograms between the urine sample and the corresponding standards (Figures S1–S4; see Supplementary Material). All of the data support the identification of the three compounds as 3HPA, HPHPA, and 3HHA, respectively, Figures 2(b), 2(c), and 2(d) graphically show the concentration distribution of urinary HPHPA, 3HPA, and 3HHA by age, respectively. The graphs clearly distinguish the ASDs cases from the controls. There is no correlation between each pair of HPHPA, 3HHA, and 3HPA (*p* values were all greater than 0.05, data not shown). Statistic results of the three compounds were summarized in Table 1. There were no statistical differences in the means for age. 3HPA (*p* < 0.001), HPHPA (*p* < 0.001), and 3HHA (*p* < 0.001) concentrations were significantly higher in ASDs children compared with age-matched controls.

### 3.3. Effect of Vancomycin on Urinary Excretions of the Three Compounds

Some studies tested vancomycin treatment for ASDs since vancomycin given orally is virtually not absorbed, and it is generally effective against gram-positive bacteria and Clostridia species [9, 16, 17]. For preventing the generation of vancomycin resistance strains, we made some modifications of vancomycin treatment. Fifty HPHPA-positive autistic children (9/50 patients 3HPA-positive and 17/50 patients 3HHA-positive) were selected for oral vancomycin treatment at standard age-appropriate dosages (50 mg/kg/d, 30 days as one therapeutic course) followed by supplement therapy with* Bifidobacterium* agent (*Bifidobacterium* BB-12, 2 pills a day). After one therapeutic course, the treatment was discontinued for 15 days and the next course began only with* Bifidobacterium* agent treatment. The subsequent* Bifidobacterium* agent treatment followed this cycle and continued depending on the severity of patients' condition assessed by Autism Behavior Checklist (ABC) and the excretions of the three compounds. Two months later, paired-sample *t*-test was applied to test the change of amounts of the three compounds before and after treatment. Significant decreases in levels of HPHPA (mean value from 302.78 to 37.06 mmol/mol creatinine, *p* < 0.001), 3HPA (from 222.30 to 15.89 mmol/mol creatinine, *p* < 0.005), and 3HHA (from 56.59 to 5.95 mmol/mol creatinine, *p* < 0.001) were found following the oral administration of vancomycin. HPHPA, 3HPA, and 3HHA were completely eliminated in 35/50, 6/9, and 12/17 cases after vancomycin treatment, respectively. Following the cessation of this treatment 3–6 months later, the concentration of HPHPA almost recovered to its initial level in 3 patients and recovered to 0.08–0.45 times their initial values in 12 patients. The levels of 3HPA and 3HHA recovered to 0.1–0.3 times their initial values in 3 patients. This may be consistent with the frequent recurrence of gastrointestinal* Clostridium* species due to germination of resistant spores following antibiotic treatment [9]. However, the recurrence was less severe compared with that in these reported studies, which may be attributed to the supplement therapy with* Bifidobacterium* agent, a probiotic bacterium that benefits the dynamic equilibrium for intestinal microecology. Furthermore, after two therapeutic course treatments, the ABC score decreased significantly (mean value from 73 to 59); 90% autistic children showed improved communication and eye contact, but no obvious improvement in stereotyped behavior was seen. These findings indicated that these compounds were probably derived from overgrown intestinal microbiota, and the pathogenesis of ASDs may be not only correlated with overgrown gut pathogenic bacteria.

Additionally, the effect of this treatment on intestinal symptoms was also studied. 32/62 children with ASDs have frequent constipation; this gut symptom was positively correlated with the HPHPA level (Pearson correlation = 0.253, *p* < 0.05). In 50 HPHPA-positive patients employed for the treatment, 22 individuals have constipation. Interestingly, after two therapeutic course treatments, 22 patients with constipation all showed remarkable improvements in constipation, which revealed that gut symptoms in ASDs may also resulted from overgrown gut pathogenic bacteria.

### 3.4. Suggested Pathway for the Metabolism of HPHPA, 3HPA, and 3HHA

**Figure 3:**
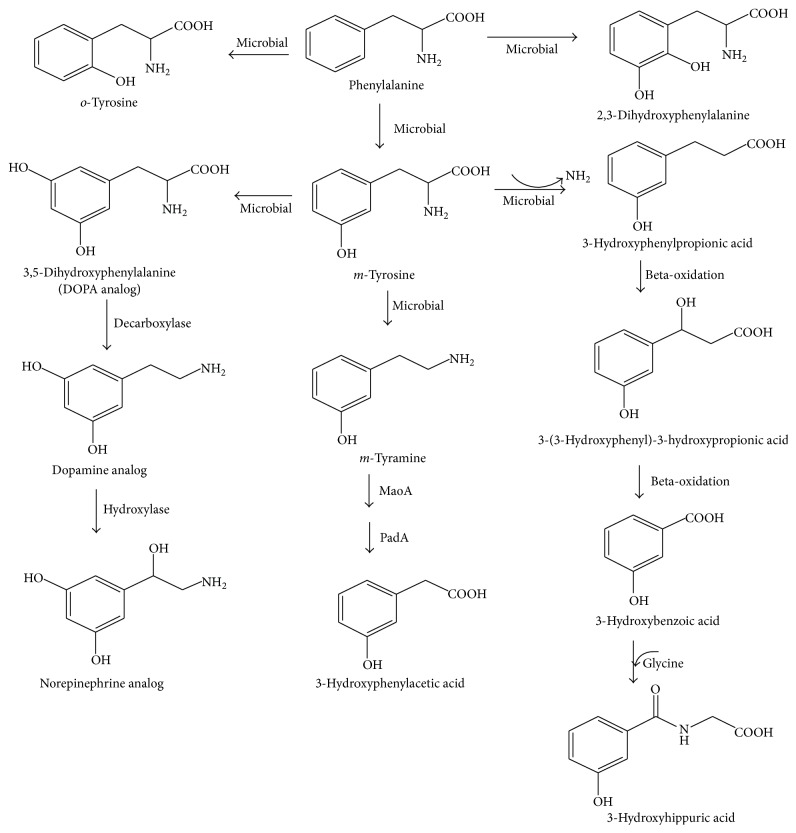
Suggested pathway for the metabolism of HPHPA, 3HPA, and 3HHA.

Based on preexisting hypothesis [9] and our experimental results, we speculated that these compounds were from disordered phenylalanine metabolism by overgrown intestinal microbiota like* Clostridium* species. As shown in Figure 3, dietary phenylalanine firstly is converted into* m*-tyrosine,* o*-tyrosine, and 2,3-dihydroxyphenylalanine by gut microbiota, for example, chloridazon-degrading bacteria [18]. It has been proved that* m*-tyrosine induces a characteristic behavioral syndrome in rats consisting of forepaw padding, head weaving, backward walking, splayed hind limbs, wet dog shakes, hyperactivity, and hyperreactivity and depletes the brain of catecholamines. Therefore,* m*-tyrosine might play a direct role in causing abnormal behaviors in ASDs [19]. It is also possible that* m*-tyrosine might form an analog of dopamine, if* m*-tyrosine is metabolized by the same enzymes that convert tyrosine to dopamine (Figure 3) [9].

*m*-Tyrosine converts to* m*-tyramine and 3-hydroxyphenylpropionic acid by decarboxylation and deamination, respectively (Figure 3). It is documented that* Escherichia coli* could induce the activities of amine oxidase (MaoA) and phenylacetaldehyde dehydrogenase (PadA) for the catabolism of aromatic amines. Phenylethylamine, tyramine, and dopamine are substrates of MaoA and PadA, leading to formation of the corresponding aromatic acids, that is, phenylacetic acid, 4-hydroxyphenylacetic acid, and 3,4-dihydroxyphenylacetic acid, respectively [20, 21]. Therefore, 3HPA would be expected to be formed if there are amounts of* m*-tyramine for the substrate of the two enzymes (Figure 3). Another metabolite from* m*-tyrosine, 3-hydroxyphenylpropionic acid, converts into HPHPA and 3-hydroxybenzoic acid in order as reported by Shaw [9]; the latter product then conjugates with glycine and forms 3HHA (Figure 3).

### 3.5. Sensitivity and Specificity

**Figure 4:**
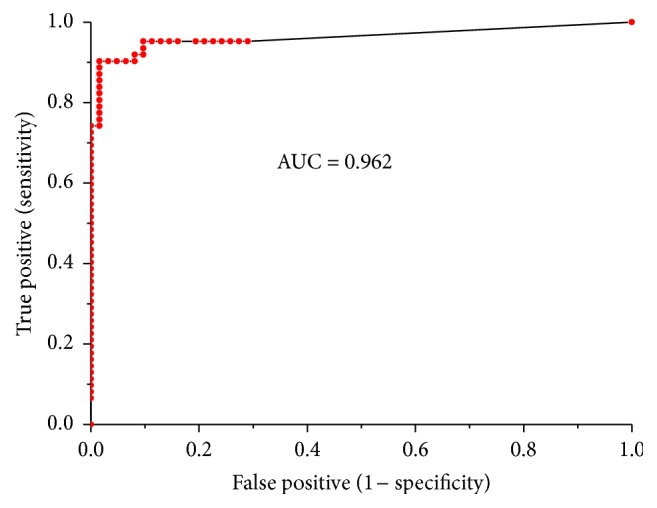
ROC curve performed by the Prediction probability based on the three metabolites. AUC value was 0.962.

Receiver-operating characteristic (ROC) analysis with sensitivity (true positives) and 1 minus specificity (false positives) of HPHPA, 3HPA, and 3HHA was used to evaluate the possibility of using these markers for diagnosing ASDs. Selected sensitivity and specificity calculations for the three metabolites measures in detecting ASDs cases are presented in Table 2. High specificity (>96%) was obtained by each metabolite for ASDs. After two-regression analysis, the optimal AUC (0.962), sensitivity (90.3%), and specificity (98.4%) were obtained by ROC curve of Prediction probability based on the three metabolites (Figure 4), which means that the three metabolites are good discriminators to differentiate between ASDs and non-ASDs control. These results indicate that the measurements of the three metabolites are strong predictors of ASDs and support the potential clinical utility for identifying a subgroup of ASDs subjects in whom disordered phenylalanine metabolism may be a salient characteristic.

## 4. Conclusions 

The present metabolomic profile approach provides comprehensive analyses of metabolites in urine and elevated levels of three aromatic acids HPHPA, 3HPA, and 3HHA were found in ASDs group compared with the controls. In particular, vancomycin has significant effect on decreasing the excretions of these compounds, which indicated that they seemed to be derived from intestinal microbiota. Further studies will have to define the degree of overlap between elevated urinary HPHPA, 3HPA, and 3HHA and intestinal microbiota composition in ASDs patients, as well as their potential relationship with gastrointestinal symptoms, abnormal behavior, and personalized response to pharmacological treatments. Additionally, the sensitivity and specificity data assessed by ROC analysis demonstrate that the measurements of the three metabolites are strong indicators of ASDs.

## Supplementary Material

Table S1 shows the urinary 96 signals, which could be auto-identified by the NIST library through comparing their fragmentation patterns composed of all the fragment ions. Peaks which could not be identified by the NIST library are not listed.
Figures S1-S4 show the further qualitative analysis of the three metabolites by comparing their retention time and fragment-ion of the chromatograms between the urine sample and the corresponding standards, respectively.

## Figures and Tables

**Table 1:** Aromatic markers found in the ASDs group and the control group.

Case status	Measure	Age (year)	HPHPA (mmol/mol creatinine)	3HPA (mmol/mol creatinine)	3HHA (mmol/mol creatinine)
Control (*n* = 62)	Frequency		15/62	1/62	4/62
	Mean	3.45	15.53	0.61	1.12
	Range	1.5–7.0	0–147.2	0–38.1	0–32.3
	Std. dev.	1.62	31.56	4.82	4.89

ASDs (*n* = 62)	Frequency		50/62	18/62	21/62
	Mean (*p* value)	3.69 (0.407)	244.18 (3.25*E* − 10)	57.99 (1.48*E* − 4)	31.02 (6.34*E* − 4)
	Range	1.5–7.0	0–1282.4	0–543.2	0–370.2
	Std. dev.	1.62	261.03	115.25	66.94

**Table 2:** Predictive values for ASDs based on the three compounds.

Test	True positive (sensitivity)	False negative (1 − sensitivity)	True negative (specificity)	False positive (1 − specificity)	Total
Criteria	HPHPA > 101.5 mmol/mol creatinine				
Control			61 (98.4%)	1 (1.6%)	62
ASDs	45 (72.6%)	17 (27.4%)			62

Criteria	3HPA > 45.2 mmol/mol creatinine				
Control			62 (100%)	0 (0%)	62
ASDs	18 (29%)	44 (71%)			62

Criteria	3HHA > 14.8 mmol/mol creatinine				
Control			60 (96.8%)	2 (3.2%)	62
ASDs	21 (33.9%)	41 (66.1%)			62

Criteria	Prediction probability > 0.65 (based on the three metabolites)				
Control			98.4%	1.6%	
ASDs	90.3%	9.7%			

## Abbreviations

ASDs: Autism spectrum disorders
ABC: Autism Behavior Checklist
AUC: Area under the curve
BSTFA: Bis(trimethylsilyl)trifluoroacetamide
TMCS: Trimethylchlorosilane
DSM: Diagnostic and Statistical Manual of Mental Disorders
GC/MS: Gas chromatography-mass spectrometry
HPHPA: 3-(3-Hydroxyphenyl)-3-hydroxypropionic acid
3HPA: 3-Hydroxyphenylacetic acid
3HHA: 3-Hydroxyhippuric acid
NIST: National Institute of Standards and Technology
PCA: Principal component analysis
ROC: Receiver-operating characteristic.
