# Supplementary material for: Urinary 3-(3-Hydroxyphenyl)-3-hydroxypropionic Acid, 3-Hydroxyphenylacetic Acid, and 3-Hydroxyhippuric Acid Are Elevated in Children with Autism Spectrum Disorders
Source: Biomed Res Int. 2016 Mar 30;2016:9485412. doi: 10.1155/2016/9485412 (PMC4829699; doi:10.1155/2016/9485412)
Supplement: Supplementary file 1 — Table S1 shows the urinary 96 signals, which could be auto-identified by the NIST library through comparing their fragmentation patterns composed of all the fragment ions. Peaks which could not be identified by the NIST library are not listed. Figures S1-S4 show the further qualitative analysis of the three metabolites by comparing their retention time and fragment-ion of the chromatograms between the urine sample and the corresponding standards, respectively. [file 9485412.f1.doc]

BioMed Research International

Electronic Supplementary Material

Urinary 3-(3-hydroxyphenyl)-3-hydroxypropanoic acid, 3-hydroxyphenylacetic acid and 3-hydroxyhippuric acid are elevated in children with autism spectrum disorders

*Xiyue Xiong, Dan Liu, Yichao Wang,Ting Zeng, Ying Peng*

*（**Maternal and Child Health Care Hospital of Hunan Province,* *Changsha 410008, China.）*

Table S1 shows the urinary 96 signals, which could be auto-identified by the NIST library through comparing their fragmentation patterns composed of all the fragment ions. Peaks which could not be identified by the NIST library are not listed.

Figures S1-S4 show the further qualitative analysis of the three metabolites by comparing their retention time and fragment-ion of the chromatograms between the urine sample and the corresponding standards, respectively.

Table S1 Urinary metabolites of the ASD group and the non-ASD control group

| Metabolites | Retention time | m/z no.1 | m/z no.2 | m/z no.3 | Match percent(%) | CAS numbers |
| --- | --- | --- | --- | --- | --- | --- |
| Lactic acid | 12.795 | 73 | 117 | 147 | 90 | 50-21-5 |
| Glycolic acid | 13.263 | 147 | 73 | 66 | 91 | 79-14-1 |
| Alanine | 13.930 | 116 | 73 | 147 | 86 | 56-41-7 |
| Glyoxylic acid | 14.626 | 73 | 147 | 218 | 84 | 298-12-4 |
| Oxalic acid | 14.714 | 73 | 147 | 130 | 87 | 144-62-7 |
| 3-Hydroxypropionic acid | 15.100 | 147 | 73 | 219 | 88 | 503-66-2 |
| Pyruvic acid | 15.305 | 73 | 147 | 232 | 91 | 127-17-3 |
| 3-Hydroxybutyric acid | 15.476 | 147 | 73 | 117 | 94 | 625-71-8 |
| 4-Hydroxybutyric acid | 15.503 | 147 | 73 | 233 | 86 | 30470-15-6 |
| Isoleucine | 15.883 | 158 | 73 | 218 | 90 | 73-32-5 |
| β-Hydroxyisovaleric acid | 16.858 | 131 | 73 | 147 | 90 | 625-08-1 |
| 2-Methylpropanedioic acid | 17.013 | 147 | 73 | 218 | 96 | 516-05-2 |
| Valine | 17.015 | 144 | 73 | 218 | 86 | 72-18-4 |
| Benzoic acid | 17.751 | 179 | 105 | 77 | 95 | 65-85-0 |
| Leucine | 18.466 | 158 | 73 | 147 | 91 | 61-90-5 |
| Proline | 19.013 | 142 | 73 | 147 | 83 | 147-85-3 |
| Isoleucine | 19.084 | 158 | 73 | 218 | 91 | 73-32-5 |
| Succinic acid | 19.417 | 147 | 73 | 247 | 93 | 110-15-6 |
| Glycine | 19.421 | 174 | 73 | 147 | 90 | 56-40-6 |
| Glyceric acid | 20.018 | 73 | 147 | 189 | 99 | 473-81-4 |
| Uracil | 20.183 | 99 | 73 | 241 | 94 | 66-22-8 |
| Fumaric acid | 20.215 | 73 | 147 | 245 | 86 | 110-17-8 |
| 5-Hydroxyhexanoic acid | 20.500 | 147 | 73 | 247 | 84 | 185956-02-9 |
| Propionylglycine | 20.690 | 73 | 159 | 188 | 91 | 21709-90-0 |
| Serine | 20.712 | 73 | 204 | 218 | 95 | 56-45-1 |
| Threonine | 21.392 | 73 | 117 | 218 | 93 | 72-19-5 |
| Isobutyrylglycine | 21.397 | 73 | 43 | 158 | 92 | 15926-18-8 |
| Glutaric acid | 21.592 | 73 | 147 | 261 | 91 | 110-94-1 |
| Methionine | 21.790 | 104 | 56 | 61 | 90 | 63-68-3 |
| 2,4-Dihydroxybutyric acid | 21.939 | 103 | 73 | 147 | 85 | 1518-62-3 |
| Butyrylglycine | 22.650 | 73 | 158 | 202 | 96 | 20208-73-5 |
| 4-Hydroxyproline | 23.265 | 73 | 140 | 147 | 86 | 51-35-4 |
| Aminomalonic acid | 23.349 | 73 | 147 | 218 | 90 | 1068-84-4 |
| Mandelic acid | 23.449 | 179 | 73 | 147 | 91 | 611-71-2 |
| Isovalerylglycine | 23.600 | 73 | 172 | 57 | 95 | 16284-60-9 |
| Erythritol | 24.096 | 73 | 205 | 217 | 83 | 149-32-6 |
| Threitol | 24.285 | 73 | 217 | 147 | 91 | 2319-57-5 |
| 5-Oxoproline | 24.496 | 156 | 73 | 147 | 91 | 98-79-3 |
| Creatinine | 25.322 | 73 | 115 | 329 | 98 | 60-27-5 |
| 2-Hydroxyphenylacetic acid | 25.449 | 73 | 147 | 253 | 94 | 614-75-5 |
| Threonic acid | 25.456 | 73 | 147 | 292 | 91 | 7306-96-9 |
| Pimelic acid | 26.063 | 73 | 125 | 147 | 85 | 111-16-0 |
| 3-Hydroxy-3-Methylglutaric acid | 26.130 | 73 | 147 | 247 | 91 | 503-49-1 |
| 3-Hydroxyphenylacetic acid | 26.270 | 73 | 164 | 147 | 99 | 621-37-4 |
| Glutamine | 26.543 | 73 | 156 | 245 | 86 | 56-85-9 |
| Arabinose | 26.659 | 73 | 217 | 204 | 85 | 5328-37-0 |
| 2-Ketoglutaric acid | 26.722 | 147 | 362 | 260 | 93 | 328-50-7 |
| 4-Hydroxyphenylacetic acid | 26.889 | 73 | 179 | 164 | 95 | 156-38-7 |
| 4-Hydroxybenzoic acid | 26.900 | 73 | 179 | 252 | 92 | 99-96-7 |
| Erythrulose | 27.170 | 73 | 147 | 103 | 92 | 533-50-6 |
| Ribose | 27.327 | 73 | 217 | 204 | 82 | 24259-59-4 |
| Fucose | 27.865 | 204 | 73 | 191 | 94 | 2438-80-4 |
| Suberic acid | 28.018 | 73 | 129 | 147 | 86 | 505-48-6 |
| Quinolinic | 28.812 | 147 | 73 | 296 | 87 | 89-00-9 |
| Xylitol | 28.875 | 73 | 217 | 103 | 91 | 87-99-0 |
| 3-Hydroxymandelic acid | 28.970 | 267 | 73 | 147 | 91 | 17119-15-2 |
| Arabitol | 28.982 | 73 | 217 | 147 | 90 | 7643-75-6 |
| Aconitic acid | 29.114 | 73 | 147 | 229 | 90 | 585-84-2 |
| Vanillic acid | 29.432 | 73 | 297 | 267 | 83 | 121-34-6 |
| Homovanillic acid | 29.600 | 73 | 209 | 179 | 99 | 306-08-1 |
| Fructose | 29.605 | 73 | 103 | 217 | 87 | 7776-48-9 |
| Azelaic acid | 29.943 | 73 | 317 | 129 | 90 | 123-99-9 |
| Shikimic acid | 30.396 | 73 | 204 | 147 | 95 | 138-59-0 |
| Ornithine | 30.584 | 142 | 73 | 174 | 93 | 70-26-8 |
| Citric acid | 30.697 | 73 | 147 | 273 | 91 | 77-92-9 |
| Hippuric acid | 30.976 | 206 | 105 | 73 | 98 | 495-69-2 |
| Methylcitric acid | 31.074 | 73 | 287 | 147 | 89 | 6061-96-7 |
| 3-(3-hydroxyphenyl)-3-  hydroxypropanoic acid | 31.132 | 73 | 267 | 174 | 96 | 3247-75-4 |
| Talose | 31.576 | 204 | 73 | 191 | 91 | 23567-25-1 |
| Xylose | 31.577 | 73 | 204 | 191 | 87 | 609-06-3 |
| Vanillomandelic acid | 31.735 | 297 | 73 | 147 | 83 | 55-10-7 |
| Sebacic acid | 31.758 | 73 | 129 | 215 | 84 | 111-20-6 |
| Tyrosine | 31.774 | 179 | 73 | 208 | 87 | 60-18-4 |
| Galactonic acid | 31.991 | 73 | 217 | 147 | 95 | 13382-27-9 |
| Histidine | 32.520 | 154 | 73 | 254 | 93 | 71-00-1 |
| Lysine | 32.524 | 84 | 73 | 156 | 97 | 56-87-1 |
| Mannitol | 32.963 | 73 | 319 | 147 | 89 | 87-78-5 |
| Glucitol | 33.098 | 73 | 205 | 319 | 91 | 6706-59-8 |
| Galactitol | 33.170 | 73 | 217 | 307 | 87 | 608-66-2 |
| Glucose | 34.151 | 319 | 205 | 147 | 87 | 921-60-8 |
| Gluconic acid | 34.272 | 73 | 147 | 333 | 87 | 526-95-4 |
| Palmitic acid | 34.358 | 117 | 73 | 313 | 95 | 57-10-3 |
| Glucuronic acid | 34.428 | 73 | 204 | 217 | 91 | 6556/12/3 |
| Galactose | 34.512 | 319 | 205 | 147 | 88 | 15572-79-9 |
| 2-Hydroxyhippuric acid | 35.026 | 73 | 206 | 324 | 92 | 487-54-7 |
| Octanamide | 35.051 | 267 | 102 | 73 | 88 | 629-01-6 |
| Myo-Inositol | 35.719 | 73 | 147 | 217 | 88 | 87-89-8 |
| Uric acid | 35.812 | 73 | 441 | 456 | 86 | 69-93-2 |
| 3-Hydroxyhippuric acid | 36.192 | 73 | 193 | 294 | 95 | 1637-75-8 |
| Tryptophan | 36.605 | 202 | 73 | 130 | 86 | 73-22-3 |
| Octadecenoic acid | 36.909 | 73 | 117 | 129 | 91 | 112-80-1 |
| 3-Indolepropionic acid | 36.968 | 202 | 73 | 147 | 96 | 180-96-6 |
| 5-Hydroxyindoleacetic acid | 37.447 | 73 | 290 | 407 | 95 | 54-16-0 |
| 4-Hydroxyhippuric acid | 37.451 | 193 | 73 | 294 | 91 | 2482-25-9 |
| Stearic acid | 37.571 | 117 | 73 | 341 | 91 | 57-11-4 |
| 2,6-Dihydroxypurine | 39.156 | 73 | 353 | 368 | 86 | 69-89-6 |


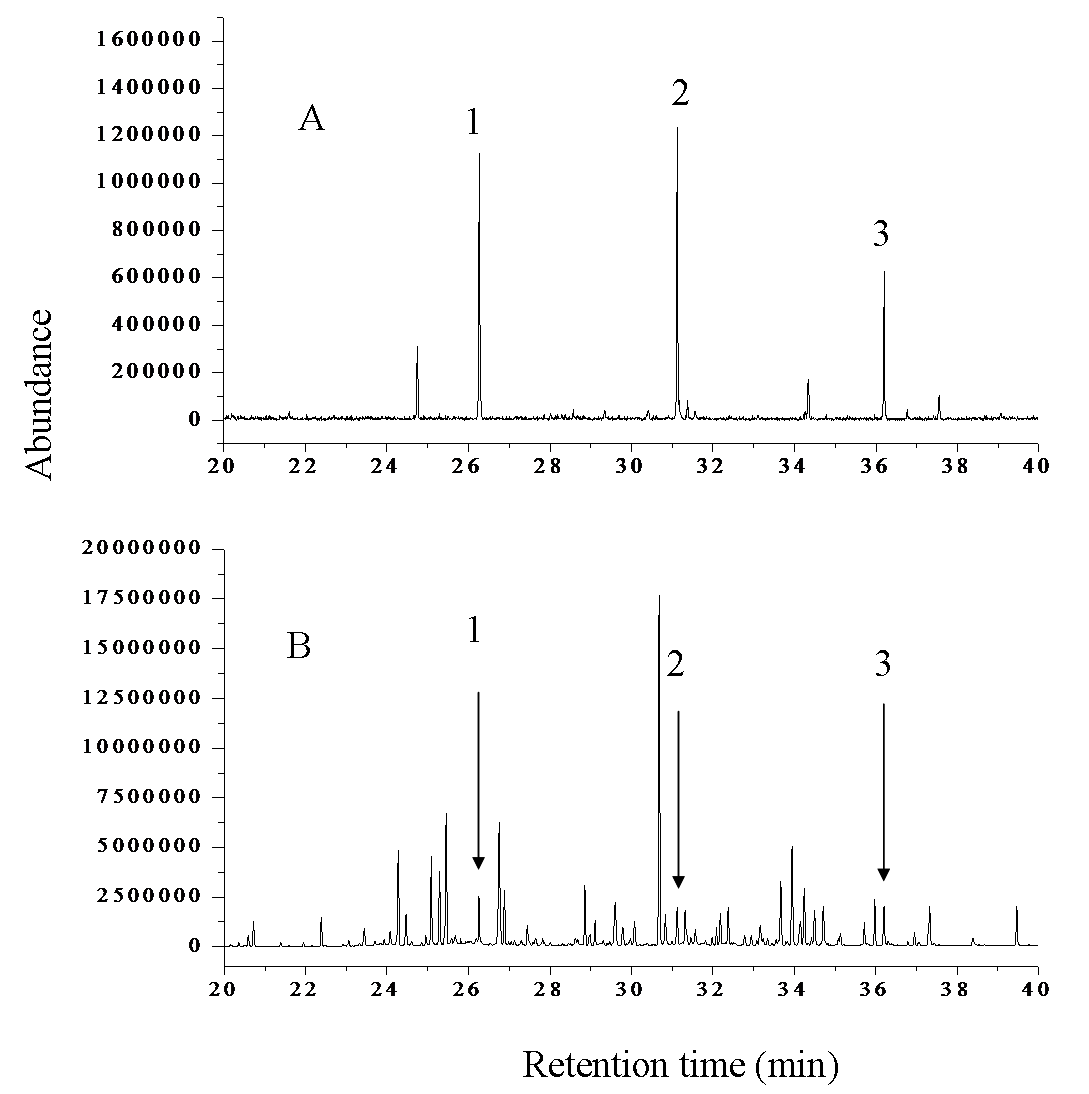


Fig. S1 Chromatograms of 3HPA, HPHPA and 3HHA in standard solution (A) and

urine sample (B). (1) 3HPA, (2) HPHPA, (3) 3HHA.


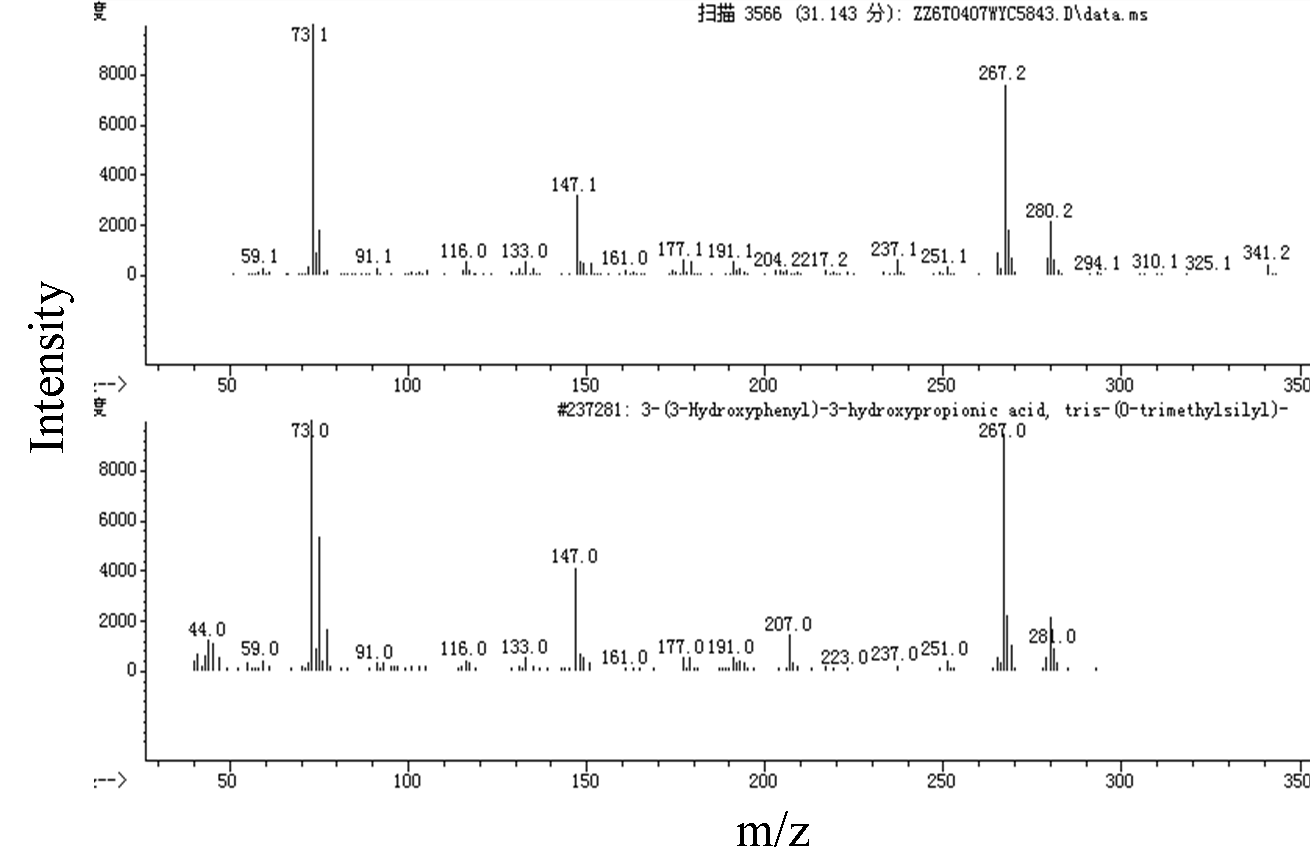


Fig. S2 Electron-impact mass spectrum of HPHPA TMS derivative.


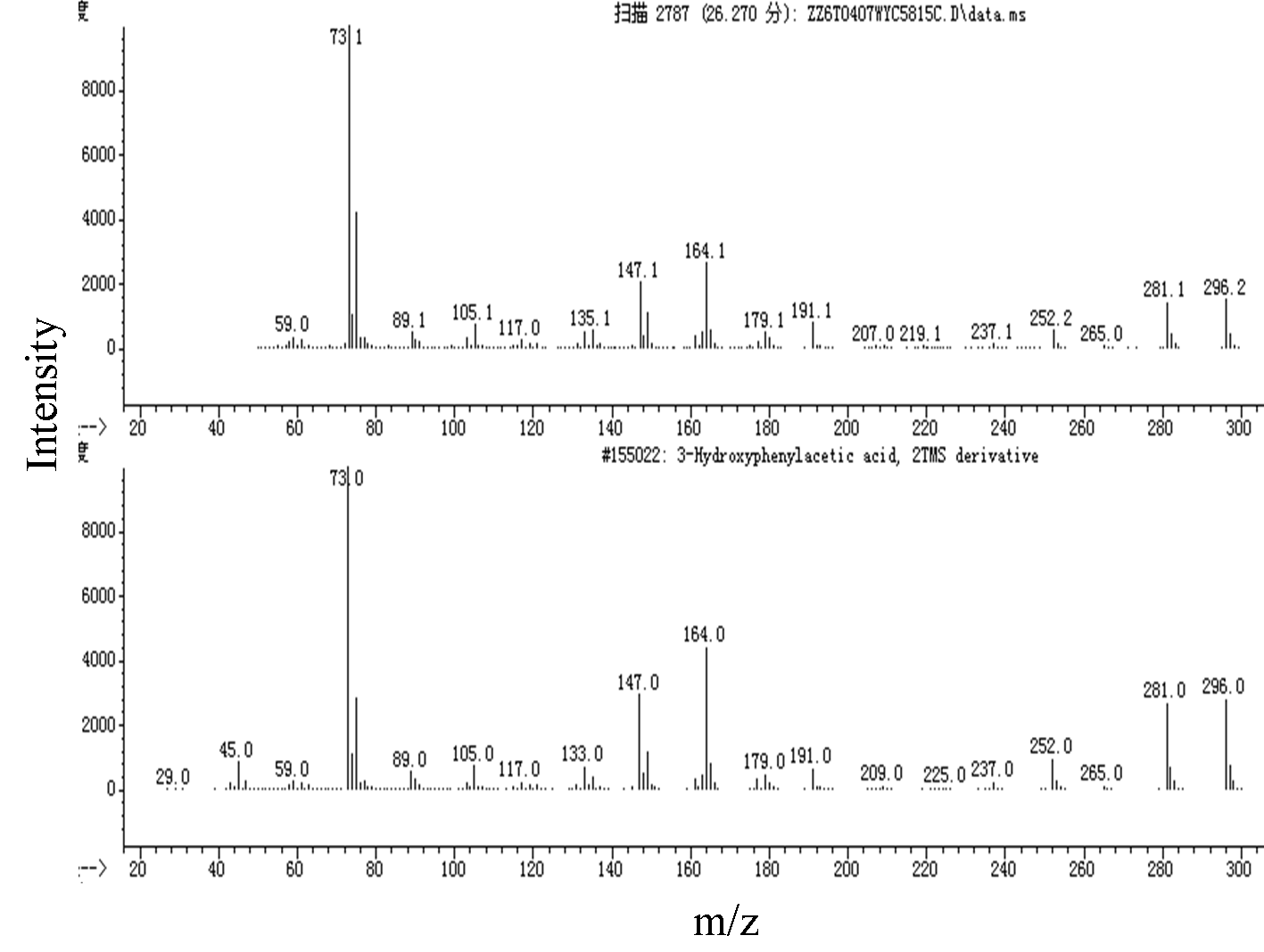


Fig. S3 Electron-impact mass spectrum of 3HPA TMS derivative.


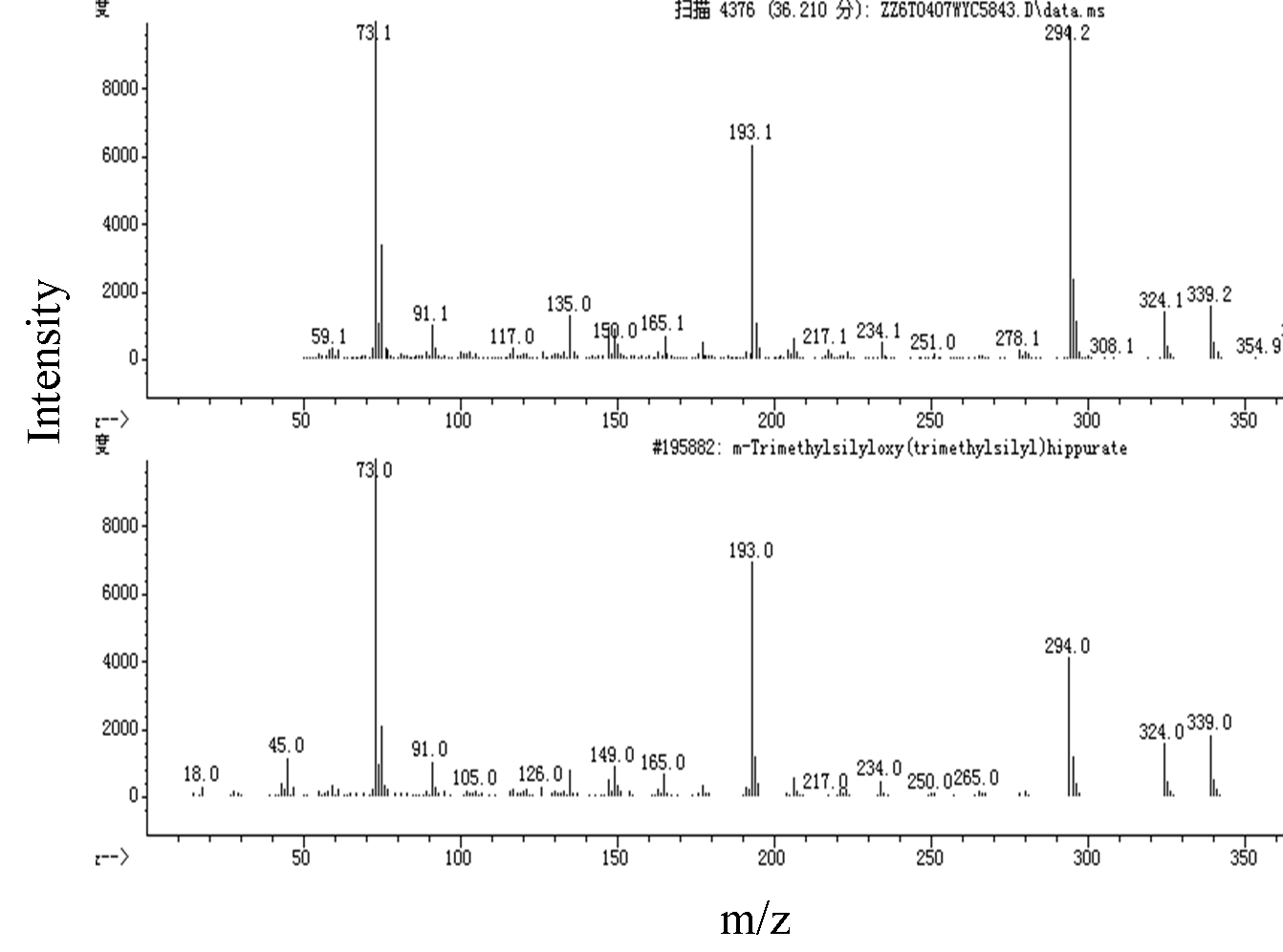


Fig. S4 Electron-impact mass spectrum of 3HHA TMS derivative.
